# Congenital Syphilis as a Measure of Maternal and Child Healthcare, Brazil

**DOI:** 10.3201/eid2508.180298

**Published:** 2019-08

**Authors:** Maria Lusia de Morais Belo Bezerra, Flávia Emília Cavalcante Valença Fernandes, João Paulo de Oliveira Nunes, Solma Lúcia Souto Maior de Araújo Baltar, Karina Perrelli Randau

**Affiliations:** Universidade Federal de Pernambuco, Recife, Brazil (M.L.M.B. Bezerra, F.E.C.V. Fernandes, S.L.S.M.A. Baltar, K.P. Randau);; Universidade Federal de Alagoas, Arapiraca, Brazil (J.P.O. Nunes).

**Keywords:** Syphilis, pregnant women, Treponema pallidum, miscarriage, stillbirth, vertical transmission, healthcare, treatment, bacteria, congenital syphilis

## Abstract

Syphilis is a sexually transmitted infection that has direct adverse effects on maternal and infant health through vertical *Treponema pallidum* transmission during early pregnancy. We evaluated congenital syphilis as a predictor of the quality of basic maternal and child healthcare in Brazil during 2010–2015. We investigated case rates and correlations with epidemiologic and socioeconomic indicators. We observed rising congenital syphilis incidence rates and increasing syphilis-associated perinatal and infant mortality rates in all regions. Case rates were highest in the Northeast, Southeast, and South, and congenital syphilis infant mortality rates were highest in the Northeast and Southeast. We observed correlations between congenital syphilis rates and infant death, spontaneous abortion (miscarriage), and stillbirth rates. We also noted correlations between rates of stillbirth caused by syphilis and inadequate prenatal care. Our study suggests gaps in basic healthcare for pregnant women and indicates the urgent need for measures to increase early diagnosis and appropriate treatment.

In 2012, ≈5.6 million new syphilis cases occurred worldwide (*1*). Syphilis is a sexually transmitted disease caused by the bacterium *Treponema pallidum* subspecies *pallidum*, which belongs to the family *Treponemataceae*. Syphilis can lead to chronic and systemic infectious disease affecting several organs, including skin and mucous membranes. Contact with genital syphilitic lesions is responsible for 95% of syphilis cases. Congenital syphilis is vertically transmitted from mother to fetus via the placental route and can cause fetal loss after the first trimester and death of the fetus or neonate. A mother can transmit the disease vertically to her newborn during delivery if she has primary syphilis lesions on her genitalia (*2*,*3*). The pathogenic potential of the bacterium is responsible for a wide range of sequelae caused by congenital syphilis (*4*).

Global epidemiologic data show *T. pallidum* caused ≈930,000 maternal infections in 2012, which led to 350,000 adverse pregnancy outcomes, including 143,000 early fetal deaths and stillbirths, 62,000 neonatal deaths, 44,000 preterm or low birthweight neonates, and 102,000 infections in infants worldwide (*5*). Progress toward elimination of maternal and congenital syphilis was observed during 2008–2012. Despite improvements, syphilis continues to affect a high number of pregnant women, causing high rates of perinatal illness and death (*5*). Elimination efforts are hindered further by a global shortage of benzathine penicillin G, a drug indicated for congenital syphilis prevention (*6*).

Countries in the Americas have experienced high rates of syphilis and congenital syphilis. Latin America and the Caribbean have demonstrated high syphilis prevalence among pregnant women (*7*), which contributes to substantial stillbirth rates (*8*). Recent data indicate that congenital syphilis rates in South America, excluding Brazil, have been stable since 2009, but Brazil has seen increasing case rates (up to 1.7/1,000 live births) that have raised the congenital syphilis rates for the continent. However, each country uses its own case definition for congenital syphilis surveillance, sometimes excluding stillbirths caused by syphilis (syphilitic stillbirths), which makes it difficult to monitor congenital syphilis case rates in the Americas (*9*).

In Brazil, Ordinance No. 542 made congenital syphilis a mandatory notifiable disease as of December 22, 1986 (*10*). Maternal syphilis, however, was not included in the national compulsory notification list until July 14, 2005, through Ordinance No. 33 (*11*). Epidemiologic surveillance demonstrates increased rates of reported congenital syphilis and deaths; infant mortality rates from syphilis increased from 2.4/100,000 live births in 2005 to 7.4/100,000 live births in 2015 (*12*). The Brazilian Ministry of Health attributes higher maternal syphilis rates to improved epidemiologic surveillance methods and expanded distribution of rapid syphilis tests. Ordinance No. 1,459 established the health policy known as the Stork Network in 2011. The Stork Network promotes improved prenatal care, increased availability of rapid syphilis tests, and fiscal subsidies to control both the maternal and congenital forms of the disease (*12*–*14*). Rapid tests use digital puncture sampling to detect the presence of treponemal antibodies and enable screening and treatment during a clinical visit. Widespread prenatal syphilis testing improved maternal treatment by reducing difficulties in patient follow-up (*15*) but simultaneously revealed the high syphilis rates in Brazil. However, a study by Cooper et al. demonstrated that this epidemic has not only become established but worsened in the country (*16*).

Implementation and expansion of the Family Health Strategy (FHS) also was intended to improve maternal and child healthcare. According to 2013 data from the National Health Survey, 53.4% of households were registered in family health units in Brazil; greatest coverage was seen in the Northeast (64.7%) and South (56.2%) and lowest coverage in the Southeast (46.0%). Expansion of the FHS for quality primary healthcare is essential for syphilis control and elimination (*17*,*18*). However, one study demonstrated inequality in healthcare access in municipalities of Brazil, low effectiveness of prenatal coverage by FHS, and no association with congenital syphilis control (*19*).

In this context, we evaluated congenital syphilis as a predictor for the quality of basic maternal and child healthcare in Brazil during 2010–2015. We assessed case rates by region and their correlation with epidemiologic and socioeconomic indicators.

## Methods

### Study Characteristics

We used a quantitative approach to conduct an ecologic study of aggregated epidemiologic data on syphilis in pregnant women and children. We collected data from the official website of the Department of Sexually Transmissible Diseases, AIDS and Viral Hepatitis of the Secretariat of Health Surveillance of the Ministry of Health in Brazil (http://indicadoressifilis.aids.gov.br). During August–November 2017, we obtained basic syphilis indicators for 6 years, 2010–2015, from the public domain database and assessed annual syphilis rates for 5 regions of Brazil: North, Northeast, Southeast, South, and Midwest. Data for 2016 were not available during the period we collected indicators for this study.

We collected data on all syphilis cases in pregnant women and those in children <1 year of age (considered congenital syphilis) that were reported to national surveillance. In Brazil, health professionals from private and public healthcare settings must complete an official epidemiologic reporting and investigation form for case notification and enter the data into the Notification of Injury Information System database of the Unified Health System. For surveillance, Brazil considers a congenital syphilis case in the following situations: live birth of any fetal age, spontaneous abortion (miscarriage) of a fetus, or stillbirth of an infant born to a woman with clinical or serologic evidence of syphilis who was not treated or received inadequate treatment, including lack of partner treatment; a person <13 years of age with increasing nontreponemal titers over time, a reactive nontreponemal titer after 6 months of age, reactive treponemal tests after 18 months of age, or a reactive nontreponemal titer higher than those of the mother; an infant or child with clinical, cerebrospinal fluid, or radiologic evidence of syphilis; or microbiologic evidence of *T. pallidum* in the placenta or umbilical cord, sample lesion, biopsy, or necropsy of a child, spontaneously aborted fetus, or stillborn infant (*12*,*20*), as outlined by the Pan American Health Organization (*9*). We considered spontaneous abortion due to syphilis as gestational loss at <22 weeks’ gestation or a fetus that weighed <500 g from a woman with syphilis who did not receive treatment or received inadequate treatment. We considered syphilitic stillbirth as death of a fetus >22 weeks’ gestation or fetus that weighed >500 g from a woman with syphilis who did not receive treatment or received inadequate treatment (*12*,*21*).

### Epidemiologic Variables

We assessed congenital syphilis by using the following variables: syphilis detection rates in pregnant women/1,000 live births; maternal schooling level; congenital syphilis cases in the absence of prenatal care; congenital syphilis cases from mothers with inadequate maternal treatment; congenital syphilis cases whose maternal partner did not receive treatment and the number of rapid syphilis tests conducted on pregnant women; congenital syphilis detection rates in children <1 year of age/1,000 live births; infant death, excluding stillbirths and fetal deaths, due to congenital syphilis in children <1 year of age/100,000 live births; spontaneous abortion due to congenital syphilis/100,000 live births; and syphilitic stillbirths/100,000 live births. We considered child death as death between birth and 12 months of age in a live-born child from a mother with syphilis, excluding spontaneous and stillbirth. We excluded data for any given variable if it was ignored or not included on the official notification form.

Because compulsory notification for congenital syphilis has been in effect since 1986, the available database is more established (*12*). We estimated congenital syphilis incidence rates by considering the number of new cases in children <1 year of age by year in a region multiplied by 1,000 and divided by the number of live births to mothers residing in the same region during that year. We used the same formula to calculate detection of maternal syphilis rates. We calculated infant mortality rates, stillbirths, and spontaneous abortions from congenital syphilis by considering the ratio of the number of cases in the region in a given year multiplied by 100,000 and the number of live births to mothers residing in that region during the same year. 

### Socioeconomic Variables

We correlated congenital syphilis cases with the following socioeconomic variables from 2010, the year of the country’s last population census: illiteracy rate, considered for persons >15 of age who cannot read or write; Human Development Index (HDI), which is a measurement calculated on the basis of income parameters (Gross Domestic Product), education (years of schooling), and health (life expectancy); Gini Index for family income per capita, which points out differences between the poorest and the richest incomes; and the Social Vulnerability Index (SVI), which is anchored in 3 dimensions: urban infrastructure, human capital, and income and work (*14*,*22*,*23*). 

### Data Analyses

We considered annual congenital syphilis rates and rate increases per region, calculated by the difference between the rates recorded in 2010 and 2015, to evaluate the temporal evolution of incidence rates. We applied the Shapiro Wilk test to verify data normality, with data adherence to the normal distribution. We applied a 1-way analysis of variance followed by a Tukey test to calculate the difference between rates, considering year and geographic region, to identify differences in pairs. We conducted a bivariate analysis by using Pearson’s correlation test to verify correlations between variables. We considered a 95% CI and significance level of 5% for all analyses. We organized the data on an Excel 2007 worksheet (Microsoft, https://www.microsoft.com) and conducted analyses by using Stata 14.0 (StataCorp., LLC, https://www.stata.com) and GraphPad Prism 5.0 (GraphPad Software, Inc., https://www.graphpad.com).

## Results

### Evolution of Congenital Syphilis Rates

We observed a continuous increase in congenital syphilis case rates in the 5 regions of Brazil during 2010–2015. Case rates doubled and, in some cases, tripled (Figure). For each year, the Northeast and Southeast had the highest congenital syphilis case rates at 2.7–6.9/1,000 live births. In 2015, case rates in the South were consistent with those in the Northeast and Southeast at 6.9/1,000 live births.

**Figure:**
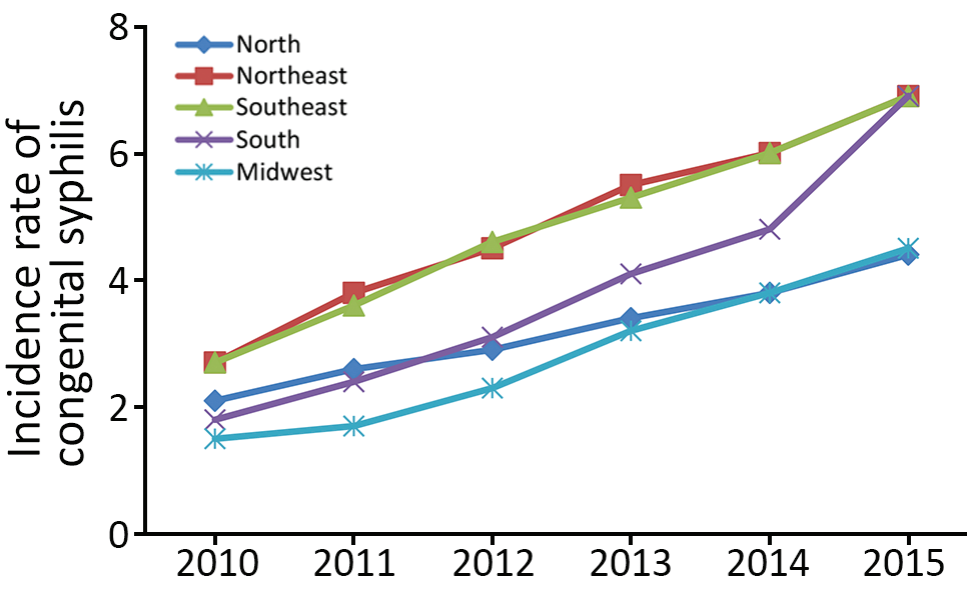
Incidence rates of congenital syphilis in children <1 year of age per 1,000 live births, by year and region, Brazil, 2010–2015.

We saw a statistically significant increase (p<0.001) in mean congenital syphilis rates per year, with the highest mean rate recorded in 2015 (5.92/1,000 live births). Congenital syphilis incidence rates increased in every triennium. Mean rates between regions were not statistically significant. We saw the highest congenital syphilis case rates in the Northeast (4.9/1,000 live births) and Southeast (4.85/1,000 live births) (Table 1).

**Table 1:** Mean congenital syphilis case rates in children <1 year of age reported to national surveillance, by year and region of Brazil, 2010–2015*

Category	No. cases, n = 77,414	Mean rate (95% CI†)		p value‡
Year				
2010	6,944	2.16^a,b,c^ (1.67–2.65)		<0.001
2011	9,484	2.82^d,e^ (2.02–3.62)		
2012	11,630	3.48^f^ (2.55–4.41)		
2013	13,967	4.30^a^ (3.33–5.27)		
2014	16,161	4.88^b,d^ (3.87–5.89)		
2015	19,228	5.92^c,e,f^ (4.69–7.15)		

Region			
North	6,055	3.20 (2.50–3.90)	0.063
Northeast	24,597	4.90 (3.62–6.18)	
Southeast	33,699	4.85 (3.56–6.14)	
South	8,974	3.85 (2.30–5.40)	
Midwest	4,089	2.83 (1.83–3.83)	
*Per 1,000 live births. Values with superscript letters indicate significant differences between the pairs calculated by using the Tukey test. †Assuming Poisson distribution. ‡1-way analysis of variance; p<0.05 indicates statistical significance.			

The mean rates for infant death, spontaneous abortion, and stillbirths per year and region increased during the period we evaluated. We detected statistically significant differences in infant death from congenital syphilis (p = 0.005) compared to the mean mortality rate for children <1 year of age. We observed a single 3-year range, 2010–2012, without statistically significant increases in infant mortality rates. We also observed statistically significant differences by region (p = 0.041); the highest mean congenital syphilis infant mortality rates were in the North (6.27/100,000 live births), Southeast (5.50/100,000), and Northeast (5.28/100,000). We did not observe statistically significant differences between death rates for the North and Midwest (Table 2).

**Table 2:** Mean infant mortality rates for congenital syphilis in children <1 year of age reported to national surveillance, by year and region of Brazil, 2010–2015*

Category	No. cases, n = 906	Mean rate (95% CI†)		p value‡
Year				
2010	90	2.74^a,b^ (1.86–3.62)		
2011	111	3.44^c^ (2.55–4.33)		
2012	147	4.22 (2.68–5.76)		0.005
2013	161	5.12 (3.37–6.87)		
2014	176	6.38^a^ (4.36–8.40)		
2015	221	6.94^b,c^ (4.94–8.94)		

Region				
North	119	6.27^d^ (3.77–8.77)		
Northeast	265	5.28 (4.16–6.41)		
Southeast	384	5.50 (4.00–7.00)		0.041
South	99	4.25 (2.82–5.68)		
Midwest	39	2.73^d^ (1.51–3.96)		

*Excluding stillbirths and fetal deaths; mean rates/100,000 live births. Values with superscript letters indicate significant differences between the pairs calculated by using the Tukey test. †Assuming Poisson distribution ‡1-way analysis of variance; p<0.05 indicates statistical significance.

We did not see statistically significant changes in rates of spontaneous abortion or stillbirth, but we did observe differences by region in the mean spontaneous abortion rates (p = 0.002) and syphilitic stillbirth rates (p<0.001). The Southeast (20.32/100,000 live births), Northeast (18.03/100,000), and South (15.02/100,000) had the highest mean spontaneous abortion rates. We also observed the highest stillbirth rates in these regions, with 24.18/100,000 live births in the Northeast, 19.63/100,000 live births in the Southeast, and 12.15/100,000 live births in the South. We observed differences in stillbirth rates between the Northeast and the South, and between the Northeast and the Midwest (Table 3). We saw increases and statistically significant differences (p = 0.046) in mortality rates associated with congenital syphilis, including death of children <1 year of age, spontaneous abortion, and stillbirth. We observed high and discrepant (p = 0.0007) rates among regions (Table 4).

**Table 3:** Mean case rates for spontaneous abortion and stillbirth from congenital syphilis reported to national surveillance, by year and region of Brazil, 2010–2015*

Category	Mean spontaneous abortion rate, n = 2,877 cases (95% CI†)	p value‡	Mean stillbirth rate, n = 3,130 cases (95% CI†)	p value‡
Year				
2010	6.72 (1.74–11.70)	0.105	7.38 (2.30–12.46)	0.154
2011	9.48 (3.54–15.42)		10.50 (2.24–18.76)	
2012	11.72 (4.74–18.70)		15.44 (7.03–23.85)	
2013	11.88 (4.20–19.56)		15.60 (6.99–24.21)	
2014	17.18 (8.54–25.82)		17.56 (11.75–23.37)	
2015	21.24 (11.34–31.14)		19.80 (14.73–24.87)	

Region				
North	5.37^a,b ^(3.19–7.54)	0.002	7.42^c,d ^(2.74–12.09)	<0.001
Northeast	18.03^a ^(14.68–21.39)		24.18^c,e,f ^(18.98–29.38)	
Southeast	20.32^b,g ^(16.82–23.81)		19.63^d,h ^(16.73–22.53)	
South	15.02 (3.65–26.38)		12.15^e ^(7.17–17.13)	
Midwest	6.45^g ^(2.81–10.08)		8.52^f,h ^(4.80–12.23)	

*Per 100,000 live births. Values with superscript letters indicate significant differences between the pairs calculated by using the Tukey test. †Assuming a Poisson distribution. ‡1-way analysis of variance; p<0.05 indicates statistical significance.

**Table 4:** Mean rates of death associated with congenital syphilis reported to national surveillance, by year and region of Brazil, 2010–2015*

Category	Mean death rate, n = 6,913 cases (95% CI†)		p value‡
Year			
2010	16.84^a^ (2.42–31.26)		
2011	23.42 (3.44–43.40)		
2012	31.38 (9.36–53.40)		0.046
2013	32.60 (10.06–55.14)		
2014	41.12 (24.12–58.12)		
2015	47.9^a^ (29.99–65.97)		

Region		
North	19.05^b,c^ (7.94–30.15)	
Northeast	47.50^b,d^ (35.52–59.48)	
Southeast	45.45^c,e^ (36.31–54.59)	0.0007
South	30.08 (7.35–52.81)	
Midwest	17.70^d,e^ (8.03–27.37)	

*Death rates include death in children <1 year of age, stillbirth, and spontaneous abortion. Values with superscript letters indicate significant differences between the pairs calculated by using the Tukey test. †Assuming a Poisson distribution. ‡1-way analysis of variance; p<0.05 indicates statistical significance.

### Variables Correlated with Congenital Syphilis

We observed positive correlations between incidence of congenital syphilis and maternal syphilis screening (r = 0.688; p<0.001), infant death due to syphilis (r = 0.679; p<0.001), spontaneous abortion due to syphilis (r = 0.859; p<0.001), stillbirths due to syphilis (r = 0.829; p<0.001), maternal schooling >8 years (r = 0.824; p<0.001), and the number of rapid syphilis tests performed in pregnant women (r = 0.519; p = 0.003). We did not observe any correlations in the other variables we investigated (inadequate maternal treatment, untreated maternal partner, no prenatal care), including socioeconomic indicators (illiteracy rate, HDI, Gini index, SVI).

We saw positive correlations between infant mortality, spontaneous abortion, and syphilitic stillbirth rates and maternal schooling >8 years and with the number of rapid syphilis test performed in pregnant women. Syphilitic stillbirth rates were the only area in which we saw correlation with lack of prenatal care (r = 0.613; p<0.001), and we did not detect any correlation between these events and socioeconomic indicators (Table 5).

**Table 5:** Correlation between congenital syphilis case rates, death, epidemiologic, and socioeconomic variables, Brazil, 2010–2015*

Variable	Congenital syphilis,† n = 77,414			Infant death,‡ n = 906			Fetal death,§ n = 2,877			Stillbirth,¶ n = 3,130	
	r value#	p value		r value#	p value		r value#	p value		r value#	p value
Maternal syphilis	0.688	**<0.001**		0.535	**0.002**		0.563	**0.001**		0.301	0.106
Infant death	0.679	**<0.001**		–	–		0.439	**0.015**		0.523	**0.003**
Fetal death	0.859	**<0.001**		0.439	**0.015**		–	–		0.814	**<0.001**
Stillbirth	0.829	**<0.001**		0.523	**0.003**		0.814	**<0.001**		–	–
Maternal schooling >8 y	0.824	**<0.001**		0.505	**0.004**		0.748	**<0.001**		0.737	**<0.001**
No prenatal care	0.305	0.101		0.132	0.486		0.360	0.051		0.613	**<0.001**
Inadequate maternal treatment	0.066	0.728		0.160	0.399		0.129	0.498		0.014	0.941
Untreated maternal partner	0.066	0.728		0.342	0.064		0.012	0.950		0.040	0.833
Rapid syphilis test of pregnant women	0.519	**0.003**		0.419	**0.020**		0.511	**0.003**		0.727	**<0.001**
Illiteracy rates**	0.476	0.418		0.430	0.470		0.251	0.684		0.348	0.566
Human Development Index**	0.355	0.557		0.456	0.441		0.013	0.984		0.040	0.949
Gini Index**	0.323	0.596		0.604	0.281		0.096	0.878		0.103	0.870
Social Vulnerability Index**	0.467	0.426		0.627	0.256		0.172	0.781		0.193	0.754

*Bold text indicates statistically significant values (p<0.05). †Congenital syphilis rates include spontaneous abortion and stillbirth rates. ‡Death in live-born infants from birth through 12 months of age. §Fetal death at <22 weeks gestation in mothers with positive syphilis seroreactive tests. ¶Stillborn infants of >22 weeks gestation born to mothers with positive syphilis seroreactive tests. #Calculated using Pearson correlation coefficient. **Values from 2010, during the country’s last population census.

## Discussion

Our study showed continuous increases in congenital syphilis incidence rates in children <1 year of age and infant death from syphilis during 2010–2015. We also observed increased rates of infant mortality, spontaneous abortion, and syphilitic stillbirth in several regions of Brazil, even with FHS expansion and a greater availability of rapid tests for the early detection of maternal disease.

Rates of primary and secondary syphilis in women are high in Brazil but are not increasing greatly over time (*21*), suggesting that the high and rising rates of congenital syphilis are due to better detection. New ordinances promoting rapid syphilis testing in pregnancy and better documentation of syphilis in pregnancy and in live and stillborn infants seem to have increased capacity to identify previously unidentified and new cases in Brazil.

Our data indicate that the congenital syphilis incidence rate in 2015 was >12 times the reduction target of <0.5 cases/1,000 live births for that year, a commitment Brazil assumed along with the Pan American Health Organization and the World Health Organization (*9*,*24*). Cooper et al. also highlighted this aspect by affirming that Brazil, despite its progress in detecting the disease, has lost its focus toward eliminating congenital syphilis (*16*).

We saw no correlation between congenital syphilis incidence rates and socioeconomic variables, including illiteracy rate, HDI, Gini index, and SVI, which counters the hypothesis that these factors contribute to the persistence of this disease. A study by Nonato et al. also showed no correlation between congenital syphilis and SVI (*25*). Data indicate social vulnerability varies between regions of Brazil (*23*), but we noted that increases in congenital syphilis rates were independent from lower or higher social vulnerability. Low social vulnerability regions, such as the Southeast and South, had high mean congenital syphilis, spontaneous abortion, and stillbirths rates. However, we also noted high mean rates in high social vulnerability regions. For instance, the North had high infant mortality rates and the Northeast had high mean rates of fetal loss after the first trimester and high mean rates of stillbirth from congenital syphilis.

Congenital syphilis is considered a sentinel event of the quality of prenatal care (*26*). We saw a correlation between a lack of prenatal care and stillbirth rates due to congenital syphilis. We also saw a correlation between congenital syphilis rates and rates of infant death, spontaneous abortion, and stillbirth. These correlations reinforce the hypothesis that congenital syphilis is a predictor of the quality of prenatal care and reveal the precarious conditions of maternal and child healthcare in Brazil. Adverse outcomes might be related to failure to diagnose or inadequately treat syphilis in pregnant women, which in turn demonstrates fragilities in basic healthcare for mothers and children. 

Nonato et al. conducted a study of 353 pregnant women in the state capital of Minas Gerais, in Southeast Brazil, and found a correlation between congenital syphilis and late prenatal care, <6 prenatal consultations, and failure to diagnose the disease during the first trimester (*25*). Our results are further corroborated in a meta-analysis by Qin et al. that found that pregnant women whose syphilis diagnosis and treatment occurred in the first trimester experienced no higher adverse outcomes than women without syphilis (*27*). In addition, that analysis showed that women whose syphilis was diagnosed and treated in the third trimester had similar adverse pregnancy outcomes to those who did not receive treatment (*27*). 

Increasing access to testing will not reduce congenital syphilis incidence if tests are conducted late in the pregnancy. Even when testing is performed early, adequate treatment is needed to produce a noticeable effect on congenital syphilis incidence. The high and increasing congenital syphilis rates we recorded might be explained by the inclusion of partner treatment in the congenital syphilis case definition, but it does not explain the higher deaths we noted.

Brazil’s Ministry of Health points out that, among mothers of children diagnosed with congenital syphilis in 2015, a total of 78.4% sought prenatal care, and 51.4% of cases were diagnosed. However, more than half of mothers (56.5%) received inadequate treatment, and 27.3% did not have access to treatment (*12*). Penicillin shortages and resistance of professional health workers to prescribe penicillin during pregnancy likely contributed to the problem. In 2015, only 55% of the basic health units in Brazil prescribed penicillin to treat syphilis in pregnant mothers. The risk for congenital syphilis increases 5-fold when maternal partners are infected (*28*), and 62.3% of maternal partners did not receive treatment in 2015 (*12*). Late identification, inadequate management, lack of partner treatment, and incomplete treatment in identified cases are important factors involved in congenital syphilis persistence (*26*). 

According to de la Calle et al., adequate treatment is essential for controlling congenital syphilis (*29*). The most effective syphilis treatment is benzathine penicillin G (*6*,*30*,*31*), but despite the medication’s efficacy and affordability, syphilis continues to be a public health problem (*2*). Benzathine penicillin G shortages have compromised treatment for diagnosed syphilis cases in several countries (*32*), including Brazil (*6*), which in turn has seen increasing congenital syphilis cases. Countries seeking to expand and improve prenatal care, reduce adverse pregnancy outcomes, and achieve congenital syphilis elimination goals should have access to a steady supply of benzathine penicillin G (*33*).

Of note, Brazil experienced increases in congenital syphilis rates before penicillin shortages. We must consider other factors that could be causing these increases, including the quality of prenatal care, resistance or difficulty of pregnant women and their sexual partners to adhere to treatment, and underreporting of the condition (*26*,*34*). Cooper et al. (*16*), as well as several others, indicate that the elimination of maternal and child syphilis transmission can only become a reality in the Americas with implementation and maintenance of clinical excellence in public health services. To reach its goal for eliminating congenital syphilis, Brazil must prioritize congenital syphilis; increase resource allocation to public health; improve syphilis screening in hard-to-reach populations; and invest in organizing health services, professional training, and review of prenatal care procedures, especially for pregnant adolescents (*2*,*35**–**38*).

Our study has some limitations. The data represent aggregated secondary data and, because we analyzed cases together and not individually, we cannot generalize the results. However, a national study demonstrated that estimates for congenital syphilis incidences at birth (3.5/1,000 live births) during 2011–2012 did not differ significantly from those registered by Brazil’s Information System for Notifiable Diseases (3.3/1,000 live births) in 2011 (*39*). Despite this finding, underreporting is a problem in Brazil (*34*,*40*). We did not exclude regional variations in case reporting because the quality of the data provided depends on technical and operational conditions of the epidemiologic surveillance system in each region. More worrisome, if underreporting exists, congenital syphilis rates could be even greater than we report.

Our study results indicate that congenital syphilis rates increased in all regions of Brazil during 2010–2015, progressing rapidly and moving the country farther from its target of <0.5 cases/1,000 live births. In addition, high rates of fetal loss after the first trimester and stillbirth rates due to congenital syphilis accentuate the seriousness of this problem. Brazil should prioritize investments in public health, especially for improving prenatal care, with a focus on the early diagnosis of maternal syphilis and strengthening management of benzathine penicillin G treatment to prevent congenital syphilis.
